# Digital Serious Games to Promote Behavior Change in Children With Chronic Diseases: Scoping Review and Development of a Self-Management Learning Framework

**DOI:** 10.2196/49692

**Published:** 2024-08-19

**Authors:** Made Ary Sarasmita, Ya-Han Lee, Fan-Ying Chan, Hsiang-Yin Chen

**Affiliations:** 1 Department of Clinical Pharmacy School of Pharmacy Taipei Medical University Taipei Taiwan; 2 Program Study of Pharmacy Faculty of Mathematics and Science Udayana University Badung Indonesia; 3 Department of Pharmacy Wan Fang Hospital Taipei Taiwan

**Keywords:** children, chronic disease, digital game, patient education, serious game

## Abstract

**Background:**

Digital serious games (SGs) have rapidly become a promising strategy for entertainment-based health education; however, developing SGs for children with chronic diseases remains a challenge.

**Objective:**

In this study, we attempted to provide an updated scope of understanding of the development and evaluation of SG educational tools and develop a framework for SG education development to promote self-management activities and behavior change in children with chronic diseases.

**Methods:**

This study consists of a knowledge base and an analytical base. This study followed the PRISMA-ScR (Preferred Reporting Items for Systematic Reviews and Meta-Analyses extension for Scoping Reviews) guidelines. To build the knowledge base, 5 stages of research were developed, including refining the review question (stage 1), searching for studies (stage 2), selecting relevant studies (stage 3), charting the information (stage 4), and collating the results (stage 5). Eligible studies that developed SG prototypes and evaluated SG education for children with chronic diseases were searched for in PubMed, Embase, Google Scholar, and peer-reviewed journals. In the analytical base, the context-mechanism-output approach and game taxonomy were used to analyze relevant behavioral theories and essential game elements. Game taxonomy included social features, presentation, narrative and identity, rewards and punishment, and manipulation and control. A total of 2 researchers selected the domains for the included behavioral theories and game elements. The intended SG framework was finalized by assembling SG fragments. Those SG fragments were appropriately reintegrated to visualize a new SG framework.

**Results:**

This scoping review summarized data from 16 randomized controlled trials that evaluated SG education for children with chronic diseases and 14 studies on SG frameworks. It showed that self-determination theory was the most commonly used behavioral theory (9/30, 30%). Game elements included feedback, visual and audio designs, characters, narratives, rewards, challenges, competitions, goals, levels, rules, and tasks. In total, 3 phases of a digital SG framework are proposed in this review: requirements (phase 1), design and development (phase 2), and evaluation (phase 3). A total of 6 steps are described: exploring SG requirements (step 1), identifying target users (step 2), designing an SG prototype (step 3), building the SG prototype (step 4), evaluating the SG prototype (step 5), and marketing and monitoring the use of the SG prototype (step 6). Safety recommendations to use digital SG-based education for children in the post–COVID-19 era were also made.

**Conclusions:**

This review summarizes the fundamental behavioral theories and game elements of the available literature to establish a new theory-driven step-by-step framework. It can support game designers, clinicians, and educators in designing, developing, and evaluating digital, SG-based educational tools to increase self-management activities and promote behavior change in children with chronic diseases.

## Introduction

### Background

Serious game (SG) educational tools that provide constructive learning with imperative goals for behavior change [1] are increasingly being applied with children with chronic diseases. Training children to self-care for their chronic diseases is highly challenging due to insufficient cognition [2], low attendance [3], complicated treatments [4], and nonadherence to treatments [5]. A properly designed SG educational tool can allow children with chronic diseases to enjoy learning how to overcome real-life challenges [6]. A fully fledged game design provides a safe and controlled environment to experience and practice self-management skills [7]. Holtz et al [8] reported that SG education had positive impacts on self-efficacy, adherence, and knowledge, which drove improvements in behavior and health outcomes over time. Directing the design and application of SGs as an educational intervention to positively support children with chronic diseases could help improve therapeutic outcomes [9].

Behavioral sciences offer insights into how to design effective SG educational tools for children with chronic diseases to achieve the dual goals of internal enjoyment and confidence while promoting their self-care abilities. To understand changes in children’s behaviors, basic principles and theories of learning, behavior, and mindset should be identified. The most commonly used theories explaining behavior change include social cognitive theory [10], self-determination theory (SDT) [11], and the mindset theory [12]. Social cognitive theory defines how behavior change can be achieved depending on personal factors, including cognition, capability, self-control, experiences, and expectations, and environmental factors, including emulation, reinforcement, and observation [10]. SDT addresses motivation and influences children to put themselves in situations in which they are exposed to SG education [13]. A growth mindset enhances greater resilience and positivity than a fixed mindset when dealing with challenges and failures [12]. Children with chronic diseases require continuous, specific self-management tasks to achieve levels of their mindset and cognitive development [12]. Incorporating behavioral theories and instructional learning into game mechanics, including practice tasks and challenges, can facilitate the changing process of a growth mindset and enhance motivation [14].

SG educational tools require a sophisticated design to avoid several potential negative consequences for children. The World Health Organization has articulated increased screen media use as a major concern due to the risk of addictive behaviors [15]. Higher gaming behavior is associated with higher levels of social, health, and behavioral problems in children and adolescents [16,17]. Playing SG interventions can promote behavior changes; however, uncontrolled excessive gaming may lead to gaming disorders [18] when games are used above the level of a child’s age and mindset [19]. It has also been reported that a large proportion of electronic games may have violent content such as fighting, hitting, destroying, and killing [19], which increases the risk of aggressive behaviors in children. Inappropriate visual designs and game elements may distract from the educational purposes [18].

An SG framework that promotes positive behavior change in children is specifically needed because children’s capabilities to respond to emotions and act when encountering difficulties differ from those of adults. Frameworks have been established for developing SG prototypes for adults [20,21]; however, only limited attention has been paid to creating a well-established theoretical SG framework for children. Children are more vulnerable to influences of digital games during their cognitive, social, and emotional development stages [2]. A stepwise SG framework is warranted to guide researchers in designing and evaluating SG educational tools for children with chronic diseases to maximize advantages and avoid unintended effects. This requires pivotal attention by researchers to creatively develop and design appropriate SG educational tools for children that balance the cornerstones of learning and playing.

### Objectives

The study purpose was to offer an updated scoping review of SG education focused on delivering self-management activities and promoting behavior change for children with chronic diseases. It provided a scope of understanding of the development and evaluation of SG educational tools and developed a systematic methodological SG-based framework for children with chronic diseases. The intended SG framework was designed according to the methodology of scoping reviews Levac et al [22] and the context-mechanism-output (CMO) approach [23]. The specific aims were to create two bases: (1) to build a knowledge base that covers all the resources required to design and develop an SG framework and (2) to construct an analytical base by integrating behavioral theories and game elements from the knowledge base to design and visualize the intended SG framework through the CMO approach. The findings of this review can benefit researchers developing and evaluating SG-based learning educational tools for children with chronic diseases.

## Methods

### Study Design

**Figure 1:**
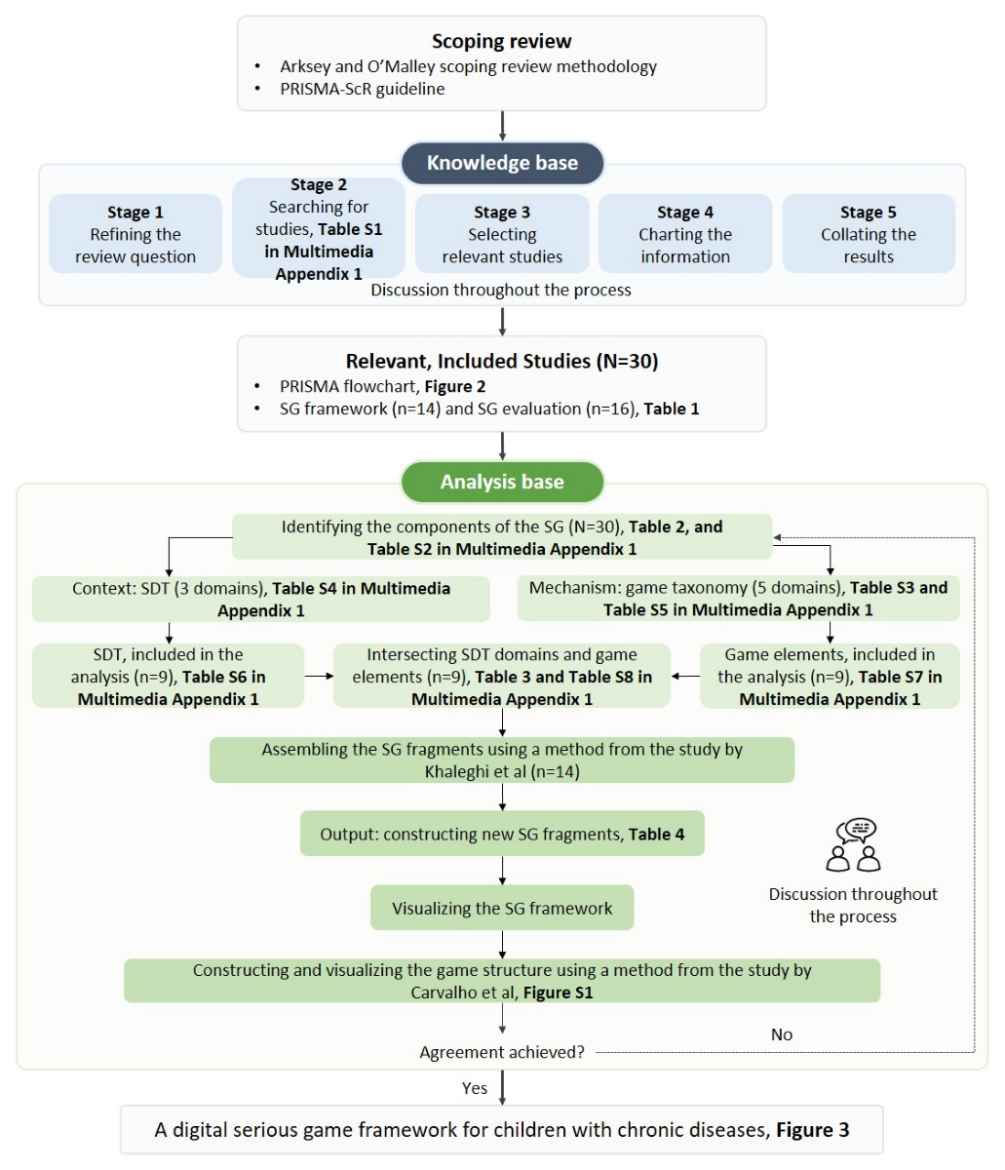
Research flowchart. PRISMA: Preferred Reporting Items for Systematic Reviews and Meta-Analyses; PRISMA-ScR: Preferred Reporting Items for Systematic Reviews and Meta-Analyses extension for Scoping Reviews; SDT: self-determination theory; SG: serious game.

Figure 1 [24-26] describes the process of developing an SG framework for children with chronic diseases using knowledge and analytical bases. This study followed the guidelines of the PRISMA-ScR (Preferred Reporting Items for Systematic Reviews and Meta-Analyses extension for Scoping Reviews) [27]. For the knowledge base, relevant studies on developing an SG framework and evaluation of SG educational tools for children with chronic diseases were searched and collated throughout 5 stages to cover all the SG resources required. The analytical base used the CMO approach and game taxonomy to build a theory-based foundation for the proposed SG framework. Relevant behavioral theories and essential game elements from relevant studies were appropriately divided into fragments, compared, and assembled to create a visualization of the proposed SG framework. Discussions were conducted throughout the study to resolve any discrepancies among researchers.

### Knowledge Base

There were five stages for building the knowledge base: (1) refining the initial question, (2) identifying relevant studies, (3) selecting relevant studies, (4) charting the information, and (5) collating the results [22].

#### Refining the Review Question (Stage 1)

SGs are digital games that blend concepts of learning and performing attitudes and are enjoyable to play, with challenging goals [28]. On the basis of the literature, we began exploring the idea of how to develop an SG framework to improve self-management and promote behavior changes in children with chronic diseases. To develop an SG framework, a scientific foundation should be built supported by documented relevant evidence.

#### Identifying Relevant Studies (Stage 2)

##### Databases and Search Strategy

Relevant studies that had developed and published established SG frameworks and SG prototypes for children with chronic diseases were searched for using electronic databases, including PubMed, Embase, and Google Scholar. A hand searching method was also used to obtain additional relevant articles in peer-reviewed journals that focused on game research and were indexed in Web of Science, such as *Games for Health Journal* and *JMIR Serious Games*. We searched for articles using keywords obtained from Medical Subject Heading terms, such as “computer game,” “video game,” and “children,” with no restriction on publication year (1980-2024). The panel in Multimedia Appendix 1 shows keyword term variations, and detailed search strategies are described in Table S1 in Multimedia Appendix 1. Reference lists of articles found through the electronic database searching were hand searched to obtain additional relevant information.

##### Inclusion Criteria

The inclusion criteria were (1) studies involving children aged 5 to 14 years, (2) studies that developed an SG framework for children with chronic diseases, and (3) studies that applied and evaluated the use of SG education for children with chronic diseases. Relevant articles were classified into two groups: (1) studies that focused on developing an SG framework and (2) studies that focused on evaluating SG educational tools for children with chronic diseases. Review or original articles explaining learning theories, behavioral theories, game theories, and game elements or presenting a general or specific SG model or framework that focused on behavior changes in children were included in the “SG framework studies” group. Randomized controlled trials (RCTs) that implemented and evaluated SG-based educational tools for children with chronic diseases were included in the “SG education studies/RCTs” group.

SG-based education is described as the use of SG prototypes or interventions, which are also known as computer games, for educational health and promotion of treatments, health education, patient adherence, therapeutic and side effect monitoring, and patient engagement. In this study, any changes in health-related outcomes in an RCT were descriptively reported. Clinical outcomes referred to any reduction in symptoms of chronic diseases and risks of complications, emergency visits, and hospitalizations. Humanistic outcomes were considered to be any condition that affected physical and social functions [29], including changes in attitudes and behaviors, adherence to treatment and medication, knowledge, quality of life, and patient satisfaction.

#### Selecting Relevant Studies (Stage 3)

The collected articles were initially imported into reference manager software (EndNote version 20; Clarivate Analysis). After removing duplicates, 2 researchers (MAS and YHL) independently assessed the articles using the inclusion criteria by examining titles and abstracts. Abstracts that met the inclusion criteria were retained for full-text review.

#### Charting the Information (Stage 4)

Characteristics of SG education for children with chronic diseases that were evaluated in RCTs were charted into a table, including authors’ information, conditions, target ages, interventions, comparators, sample sizes, study duration, length of the study, and health-related outcomes. Data were summarized by 2 authors (MAS and YHL). Disagreements were resolved through discussion involving a third reviewer (HYC).

#### Collating the Results (Stage 5)

The components of SGs were collated from the included studies using the CMO approach [23]. According to the CMO approach, “context” consists of any fundamental principles that enhance the efficacy of SG education to induce behavior changes, including behavioral theories, learning theories, and game theories. “Mechanism” refers to rules of how a game works, the dynamics through which children interact in response to the game, and the game’s appearance. It includes game elements to actively engage and motivate target users to perform self-management activities and positive behaviors. “Output” is related to any outcomes, study output, or study prototype.

### Analytical Base

#### Identifying SG Components

Components of SG educational tools comprise behavioral theories, learning theories, game theories (context), and game taxonomy (mechanism). Behavioral theories and game elements were identified after collating all the included studies. Details of the identified SG components based on the CMO approach are presented in Tables S2 and S3 in Multimedia Appendix 1. The most often used behavioral theories were selected for inclusion in the analysis (n=9), as shown in Table S4 in Multimedia Appendix 1. Detailed categories of the game taxonomy are shown in Table S5 in Multimedia Appendix 1. Table S4 in Multimedia Appendix 1 presents SDT domains, consisting of the psychological needs of autonomy, competence, and relatedness to boost motivation. The autonomy domain refers to how users make decisions and boost the sense of control, such as adjusting choices, levels, and difficulties [11]. The sense of control evokes autonomy and fuels users’ willingness to continue playing. Competence refers to achieving targeted goals of successful actions, such as challenges, learning tasks, competitions, and rewards [11]. Relatedness expresses how children interact and how their interactions affect others within the game, such as avatars, feedback, and emotions [11]. Game taxonomy was applied to identify game elements in all studies, including social features, presentation, narrative, identity, rewards and punishments, and manipulation and control [30]. A total of 3 domains of SDT and 5 categories of game taxonomy intersected based on similar characteristics. Those elements were reintegrated to build new, appropriate game elements for children with chronic diseases. These SG components are reviewed and discussed throughout the analysis.

#### Assembling SG Fragments and Visualizing an SG Framework

SG fragments describe the strategies or systematic procedures for designing, developing, and evaluating SG educational tools for children with chronic diseases. After identifying SG components through SDT and game taxonomy, 2 researchers (MAS and YHL) individually analyzed SG framework studies (n=14) based on their step-by-step procedural techniques (fragments). The collected fragments were appropriately assembled into 5 steps following the method by Khaleghi et al [24]: objective definitions (step 1); users’ needs and game element identification (step 2); game concept generation, game mechanics selection, and prototyping (step 3); implementation (step 4); and monitoring (step 5). Fragments of SG development were appropriately reintegrated to build a new SG framework.

Fragments for building up a new SG framework were established and consisted of 3 main phases (requirements, design and development, and evaluation). Each main phase was redesigned to formulate 2 procedural steps and generate output materials. A proposed SG framework was visualized by one researcher (MAS) and then carefully reviewed by 2 other authors (YHL and HYC). Following the method by Carvalho et al [25], a game structure was designed to supplement the SG framework to explain the actions, tools, and achieved goals of learning and gaming (Figure S1 in Multimedia Appendix 1). The process of assembling the SG fragments and then visualizing the SG framework and game structure were discussed among the 3 researchers throughout the study.

## Results

### Knowledge Base

#### Overview

**Figure 2:**
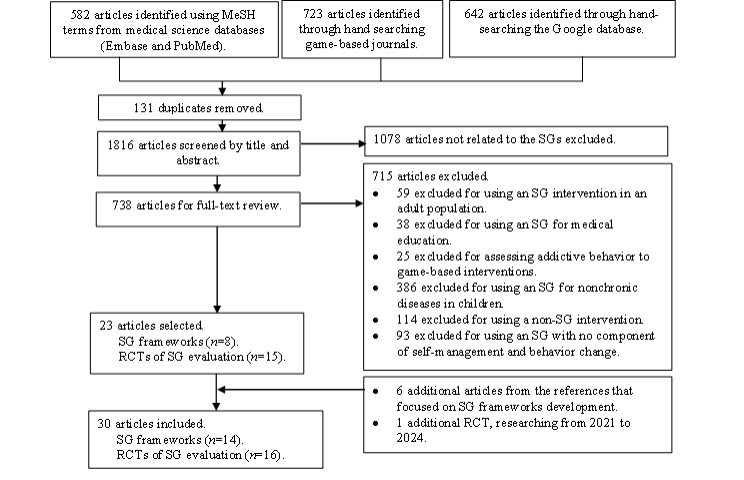
The PRISMA (Preferred Reporting Items for Systematic Reviews and Meta-Analyses) flow diagram. MeSH: Medical Subject Heading; RCT: randomized controlled trial; SG: serious game.

We retrieved 1947 articles from PubMed (n=451, 23.16%), Embase (n=131, 6.73%), Google Search (n=512, 26.3%), Google Scholar (n=130, 6.68%), *JMIR Serious Games* (n=272, 13.97%), and *Games for Health Journal* (n=451, 23.16%). After removing duplicates, 738 full-text articles were reviewed. In total, 30 articles were included in the scoping review, consisting of 16 (53%) RCTs that evaluated SG educational tools for children with chronic diseases and 14 (47%) studies on SG frameworks (Figure 2).

#### Charting the Information

Table 1 describes the included 16 RCTs that evaluated the use of SG educational tools for children with chronic diseases. SG educational tools that blended the concepts of learning and gaming were developed for children with asthma (7/16, 44%) [31-37], obesity and risk of diabetes or only diabetes (4/16, 25%) [38-41], cancer (2/16, 13%) [42,43], cystic fibrosis (1/16, 6%) [44], cerebral palsy (1/15, 6%) [45], and HIV or AIDS (1/15, 6%) [46]. The number of study participants ranged from 10 to 375; however, 60% (9/15) of the studies had <100 participants, as shown in Table 1. SG educational tools were delivered to children whose ages ranged from 3 to 17 years, with an average duration of play of approximately 40 minutes.

A total of 47% (7/15) of the studies reported improvements in clinical outcomes, including improved energy expenditure, heart rate, and blood pressure [31], or reduced symptoms, such as dyspnea and fatigue [31-33,44], as well as fewer hospitalizations [32] and unscheduled physician visits [38]. Regarding humanistic outcomes, 87% (13/15) of the RCTs evaluated behavioral outcomes. In total, 47% (7/15) of the studies presented improvements in knowledge, and 40% (6/15) reported improvements in behaviors, including asthma self-management [32,33], healthy dietary habits [39], communication with parents [38], disease-related risk communication [46], and lower medication use [34]. A total of 100% (15/15) of the studies evaluated users’ acceptance and satisfaction, resulting in 100% (15/15) of the RCTs showing positive acceptance toward SG educational tools and consideration of SG educational tools as enjoyable strategies for learning and practicing self-management tasks. None of the 15 RCTs evaluated economic outcomes.

**Table 1:** Summary of the included serious game educational tools in randomized controlled trials (N=16).

Study, year	Condition	Age of users (y)	Intervention	Control	Sample size, N	Health outcomes
Rubin et al [33], 1986	Asthma	7-12	Asthma Command	Nonasthma computer game	65	Lower asthma-related acute visits, improved asthma knowledge, and improved asthma management–related behaviors
Bartholomew et al [32], 2000	Asthma	7-17	Watch, Discover, Think, and Act	Usual care	133	Fewer symptoms and hospitalizations, increased asthma knowledge, improved correct decision-making in the game scenario (62%) and engagement in the game (84%), and children felt satisfied (97%)
Yawn et al [37], 2000	Asthma	3-12	Air Academy	Usual health education	87	Improved asthma knowledge, and children and teachers felt satisfied
Huss et al [34], 2003	Asthma	7-12	Wee Willie Wheezie	Written asthma education	101	Children felt that the game could have been more esthetic.
Shames et al [36], 2004	Asthma	5-12	Bronkie’s Asthma Adventure	Usual care and video game	119	Increased asthma knowledge, and children had a high interest in the program.
McPherson et al [35], 2005	Asthma	7-14	The Asthma Files	Asthma booklet	101	Lower oral steroid use, improved asthma knowledge, improved internal locus of control, high interest in the program (35/37, 95%), children felt that the game helped them gain asthma knowledge (32/37, 87%), and fewer school absences
Gomes et al [31], 2015	Asthma	5-9	Reflex Ridge from The Kinect Adventures Program	Treadmill session	36	Lower level of FeNO^a^, improved asthma control and exercise capacity, higher energy expenditure, higher motivation, and high endurance with the game
Salonini et al [44], 2015	Cystic fibrosis	8-17	The Kinect Adventures Program	Stationary cycle training	30	Less frequent dyspnea and fatigue and high enjoyment of the game
Kato et al [43], 2008	Cancer	13-29	Remission	Noncancer computer game	375	Improved adherence to the use of cancer medications, increased self-efficacy, and greater knowledge
Hamari et al [42], 2019	Cancer	3-16	Nintendo WiiFit	Usual care	36	High acceptability and participation (77%), but the game was not followed as recommended.
Brown et al [38], 1997	Diabetes	8-16	Packy and Marlon	Video games with no health education content	59	Improved self-efficacy, better self-care behavior, increased diabetes-related communication with parents, and fewer unscheduled visits to the physician
Baranowski et al [40], 2011	Obesity and risk of diabetes	10-12	Escape from Diab and Nanoswarm	Knowledge-based internet game	133	Increased habit of eating healthy foods and high enjoyment of the game (80%-90%)
Baranowski et al [39], 2019	Obesity and risk of diabetes	10-12	Escape from Diab and Nanoswarm	Knowledge-based internet game	200	Increased expectations for gameplay
Weiland et al [41], 2022	Obesity and risk of diabetes	9-12	Kids Obesity Prevention or family intervention	Child intervention	23	Increased knowledge gain in children and parents, maintenance of knowledge in parents, and high acceptance of the game
Winskell et al [46], 2018	HIV or AIDS	11-14	Tumaini	Usual care	60	Improved sexual health–related knowledge, greater self-efficacy, and improved intention for risk avoidance strategies and sexual risk communication
Pin and Butler [45], 2019	Cerebral palsy	6-14	Interactive game	Usual care	18	High enjoyment of the game

^a^FeNO: fractional exhaled nitric oxide.

#### Collating SG Components

Table 2 describes essential components of the SG-based education for developing the proposed SG framework based on the CMO approach (N=30). For context, several studies applied behavioral theories (23/30, 77%) and game theories (22/30, 73%). Regarding mechanisms*,* several aspects were concerned with embedding social features or feedback (28/30, 93%); presentation or esthetics (30/30, 100%); personalization, including narratives (24/30, 80%), characters (23/30, 77%), and rewards and punishments (26/30, 87%); and manipulation and control, including game genre or rules (23/30, 77%), game goals (28/30, 93%), and challenges (27/30, 90%). Regarding output, 27% (8/30) of the studies generated specific SG frameworks for children with chronic illnesses, including children with diabetes [39,40,47,48], children with cystic fibrosis [49], and children who needed physical rehabilitation [24,50,51]. The most commonly used behavioral theory in the studies was SDT (9/30, 30%). Relevant studies that used SDT as the behavioral theory foundation are identified in Table S6 in Multimedia Appendix 1. Game elements from all the included studies (N=30) are described in Table S7 in Multimedia Appendix 1.

**Table 2:** Collation of serious game (SG) components based on the context-mechanism-output approach (N=30).

				SG framework studies (n=14), n (%)		Randomized clinical trials (n=16), n (%)		Total studies, n (%)
**Context**								
	Behavioral theories			12 (86)		11 (69)		23 (77)
	Game theories			13 (93)		9 (56)		22 (73)
**Mechanism**								
	Social (feedback)			13 (93)		15 (94)		28 (97)
	Presentation (esthetic)			14 (100)		15 (94)		29 (100)
	**Personalization**							
		Narrative (story and narrative)	13 (93)		11 (69)		24 (83)	
		Identity (characters and avatars)	12 (86)		11 (69)		23 (79)	
	Rewards and punishments			13 (93)		13 (81)		26 (90)
	**Manipulation and control**							
		Game genre and rules	10 (71)		13 (81)		23 (79)	
		Game goals	14 (100)		15 (94)		29 (97)	
		Challenges	12 (86)		15 (94)		27 (93)	
**Output**								
	**Health outcomes**							
		Behavioral outcomes	—^a^		14 (88)		14 (47)	
		Learning outcomes	—		12 (75)		12 (40)	
		Clinical outcomes	—		11 (69)		11 (38)	
	**Dissemination of the SG framework**							
		A specific framework for children	8 (57)		—		8 (28)	
		General framework	6 (43)		—		6 (21)	

^a^Not applicable.

### Analytical Base

#### Identifying SG Components

Table 3 summarizes the intersection of the 3 domains of SDT and 5 categories of game taxonomy (n=9). On the basis of our findings, game elements that should be inserted in a proposed SG framework for children with chronic diseases include feedback, such as tailored messages and links to social media (social); visual designs, such as images, videos, animations, and cartoons, and audio designs, such as music and sounds (presentation); avatars, characters, and emotions (identity); storyline (narrative); rewards and progress bar (rewards and punishments); and challenges, choices, competitions, goals, rules, levels, and tasks (manipulation and control). Details of the intersection of SDT and game taxonomy (n=9) are described in Table S8 in Multimedia Appendix 1.

**Table 3:** Intersection of the domains of self-determination theory and game taxonomy for the proposed serious game (SG) framework.

Self-determination theory of the proposed SD framework	Game taxonomy				
	Social features	Presentation	Narrative and identity	Rewards and punishments	Manipulation and control
Competence	—^a^	Educative materials [50,51]: learning content and learning instructions	—	Rewards [21,24,39,40,47-51]: points, progress bar, badges, and stars; punishments: NR^b^	Challenges [21,24,39,40,47-51]: competitions [21,24,39,40,48,50,51], levels [21,24,39,40,48,50,51], tasks [21,47,50,51], game rules [24,39,40,50], and goals [24,39,40,47,50]
Autonomy	—	Presentation: visual design [21,24,39,40,47-51]: images, videos, animations, cartoons, and attractive layout; audio design [24,39,40,47-51]: music and sounds	—	—	Choices [24,39,40,47,50,51] and difficulty adjustment [24,39,40,49,50]
Relatedness	Feedback [21,24,39,40,47-51]: tailored messages [39,40,48] and social media [21,49]	—	Narrative [21,24,39,40,47,51]: storyline; identity [21,24,39,40,48-51]: avatars, characters, and emotions	—	Motivation [47]

^a^Not applicable.

^b^NR: not reported.

#### Assembling SG Fragments and Visualizing the Framework

Table 4 presents the determination of SG fragments from the included studies (14/30, 47%) and then assembles those SG fragments into a proposed SG framework. Each existing study offered different procedural steps for developing an SG prototype, yet the game elements and behavioral theories complemented each other. Only 50% (7/14) of the studies created SG prototypes [24,25,40,47,49,52,53]. Of the 14 SG framework studies, 3 (21%) specifically targeted self-management activities [21,41,47]. On the basis of these findings, we reintegrated those fragments into 3 main phases with 6 step-by-step procedural techniques.

In phase 1 (requirements), there are 2 important steps, including exploring the idea and SG requirements (step 1) using literature reviews and identifying target users’ needs (step 2) using iterative discussions or interviews. The outputs of phase 1 are relevant theories, game taxonomy, and children’s needs and preferences. In phase 2 (design and development), 2 steps should be considered by designers, including designing the game elements and educative materials (step 3) and building an SG prototype (step 4) using appropriate software programs and hardware equipment. The output of phase 2 is the SG prototype with a game structure. The final phase is phase 3 (evaluation), which is concerned with evaluating the SG prototype using a clinical trial design (step 5) and marketing the SG and monitoring its use (step 6) throughout clinic-based practice. Outputs of phase 3 are health outcome results and the final SG prototype with recommendations for its use.

**Table 4:** Assembling serious game fragments from the selected studies (N=14).

Study, year	Step 1 (objective definition)	Step 2			Step 3			Step 4 (implementation)		Step 5 (monitoring)
		Users’ needs	Game element identification	Game mechanic		Prototyping				
AlMarshedi et al [21], 2016	+^a^	NR^b^	+	+		NR	NR		NR	
Baranowski et al [48], 2011	+	+	+	+		+	+		+	
Carvalho et al [25], 2015	+	+	+	+		+	+		+	
Beristain-Colorado et al [50], 2021	+	NR	+	+		NR	NR		NR	
Dörrenbächer et al [51], 2014	+	+	+	+		+	+		+	
Epstein et al [23], 2021	+	+	+	+		NR	NR		NR	
Hansen [49], 2017	+	+	+	+		+	+		+	
Jaccard et al [54], 2021	+	NR	+	+		NR	NR		NR	
Khaleghi et al [24], 2021	+	+	+	+		+	NR		NR	
Mummah et al [20], 2016	+	NR	+	+		NR	NR		NR	
Starks [52], 2014	+	+	+	+		NR	NR		NR	
Thompson et al [47], 2010	+	+	+	+		+	+		+	
Verschueren et al [53], 2019	+	+	+	+		+	+		+	
Wattanasoontorn et al [55], 2013	+	NR	+	+		NR	NR		NR	

^a^Present or reported.

^b^NR: not reported.

#### The Proposed SG Framework

##### Overview

**Figure 3:**
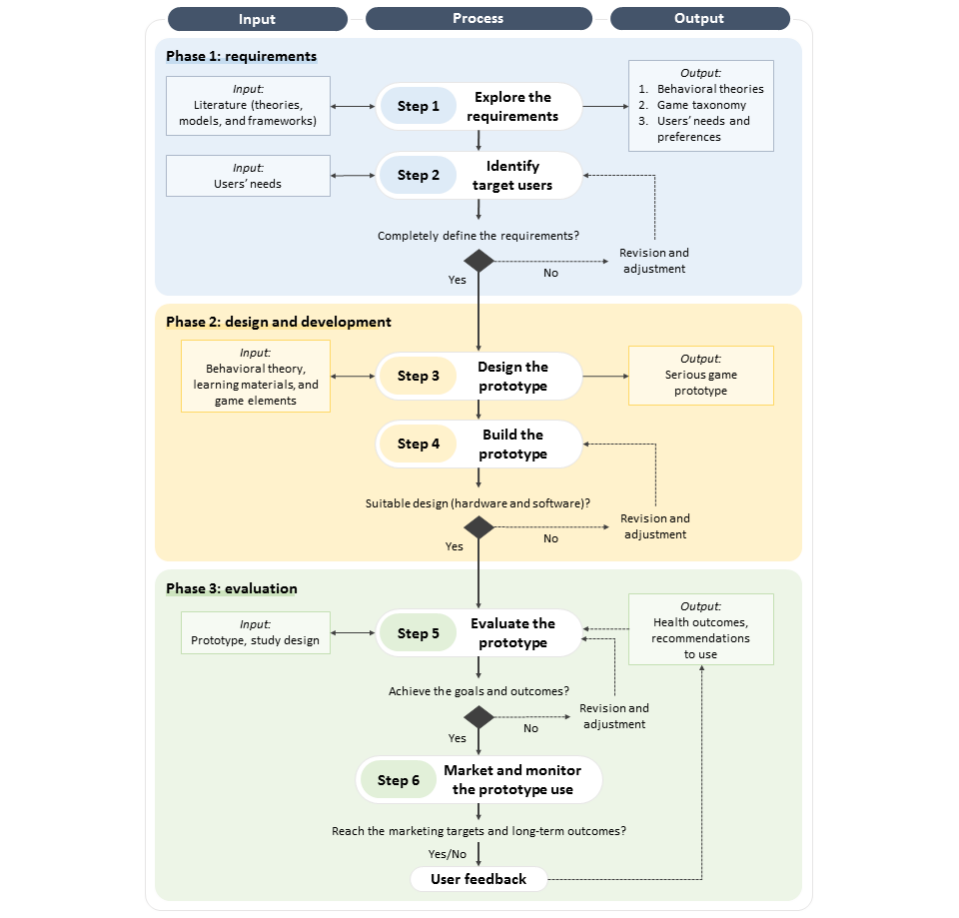
The proposed Self-Management Interactive Learning and Entertainment framework.

On the basis of our knowledge base and analytical base, we propose a new SG-based framework that integrates the principles of SDT and game elements into self-management practices, titled Self-Management Interactive Learning and Entertainment for children with chronic diseases, as presented in Figure 3. It consists of 3 main phases, starting from the requirements of SG educational tools (phase 1), design and development (phase 2), and evaluation of the SG educational tools (phase 3). In total, 2 procedural steps are included in each phase, resulting in 6 procedural steps. Each step has input materials as the foundation to support the actions and process and to produce output materials. Output materials in the first phase (phase 1) can be used as the input for the next phase (phase 2), and so forth. Each phase has critical points, revisions, and adjustments that should be considered by any game designer, researcher, or health care provider who would like to create an SG educational tool. Each game should be suitable for target users and their conditions; for example, an SG educational tool for children with asthma should have specific learning materials (asthma action plans and asthma medications), target goals (improved quality of life), and tasks and challenges (asthma self-management activities, breathing technique, and proper asthma medication use) that might differ from those of other diseases. Figure S1 in Multimedia Appendix 1 shows the gaming and learning structure of an SG prototype that blends SDT domains and game elements. The mechanism of how players achieve the target goal by accomplishing challenges should be set in clear game rules. Children will make their first choice by selecting an avatar or character, directly engaging with the game. Learning materials will help children understand their disease management, yet the game instructions will help them simultaneously observe challenges and tasks. After completing the tasks, their performance should be rewarded through points or a performance meter.

##### Exploring SG Requirements (Step 1)

A robust theoretical SG-based foundation should be established using literature reviews that gather principles of learning theories, behavioral theories, and game theories. This step is aimed at exploring SG requirements by searching for evidence related to game-based behavior change programs for children using electronic databases, for example, behavioral and game theories. If such evidence is not available, it is recommended to consult established game developers and collect perspectives from target audiences regarding obstacles in their daily lives [21]. According to Bramer et al [56], critical points include how to determine a clear and focused research question, how to choose databases and interfaces to begin, how to use an appropriate search technique, and how to document and translate collected documents. After determining relevant SG literature, game elements and behavioral theories should be translated and adopted for use by children with chronic diseases. Relevant articles can be used as inputs to conduct iterative discussions to identify users’ needs.

##### Identifying Target Users (Step 2)

Step 2 began through iterative discussions with a multidisciplinary, collaborative team. The iterative approach refers to the iterative process of refining, creating, and revising a project until agreement is achieved, and it is commonly used for agile software development [57]. The aim of this step is to collect perspectives on identifying users’ profiles and needs, their daily difficulties and barriers related to their chronic conditions, and target outcomes [7]. In this step, critical points emphasize what the players’ backgrounds are, what age groups are considered, to which chronic conditions would the SG educational tool be applied, how many users would be involved in the game, and what outcomes need to be achieved [53]. A multidisciplinary team consisting of pediatricians, child psychologists, child educators, game prototype designers, and multimedia experts [54] needs to identify resilient attitudes and consistent stimuli that suit children’s characteristics. Designers should carefully identify users’ cultures, beliefs, mindset, and literacy to concisely adopt those preferences into the game’s elements [24]. Directly involving children through focus group discussions or in-depth interviews will help the team gamify self-management tasks based on their needs and level of understanding, including medication adherence, physical exercise, and maintenance of healthy dietary habits.

##### Designing the SG Prototype (Step 3)

A key driver for successful SG education is consolidating a balance between self-management tasks (serious) and game elements (entertainment) [6,58]. This step aims to design the mechanism and user interface of the game itself by consolidating the most appropriate behavioral theories, learning materials, and game elements. Designers begin to create a prototype after establishing selected relevant theories, game elements, and users’ needs and outcomes (input). First, designers should elucidate selected, well-established behavioral and learning theories into educational materials and game taxonomy into appropriate game elements for children. Game designers should consider several critical points, including what topics are inserted into the learning materials, which game elements are best suited to achieve the desired outcomes, and how interacting with the game can lead to targeted behaviors [49]. The educational materials should contain disease information, including its pathophysiology, signs and symptoms, treatments and medications, self-management, side effects, the importance of adherence, and daily practices.

It is recommended to insert game elements that offer enjoyment to stimulate children to play, at the same time directly motivating them to learn [51]. Cartoon characters, genres, and stories represent personalization for children [52]. To grow children’s mindset, challenges should be designed with competitive levels and rewards provided when a mission is accomplished [12]. A role model with a positive attitude should be inserted into the SG design to encourage children to become masters of practicing self-management activities. Adding these elements facilitates children responding when confronted with conflicts [50] and enhances their sense of resilience. It is important to design an SG prototype that mimics real-world circumstances by setting precise goals and instructing players to perform targeted skills over time [25]. It is also suggested to embed the features of feedback, a progress bar, or trend alerts to evaluate their performance after completing the challenges.

##### Building the SG Prototype (Step 4)

Step 4 aims to develop an actual prototype based on the selected behavioral theories and game elements. It requires extensive discussions with researchers, multimedia experts, and the game industry to integrate the technology into a game console. A graphic user interface should be built to present the set of game rules. Designers may consider facilitating level adjustments if children fail to win to maintain the developed mindset. Critical points are how the prototype can be built for efficient learning and playing, how to perform such tasks, and how to rapidly respond regarding those performances. Esthetics is an essential aspect to be considered. Game visuals can be appropriately created using 2D or 3D formats [55]. Music and animation can be added to enhance excitement and enjoyment. To effectively promote self-management tasks, virtual reality SGs should be equipped with body movement tools that specifically target childhood chronic diseases that involve physical disabilities [50]. Moreover, privacy should be protected because SG prototypes can be used in multiplayer settings, and the accessibility to enter measured data should be restricted [13].

##### Evaluating the SG Prototype (Step 5)

Step 5 focuses on evaluating the efficacy of the SG educational tools and unexpected effects after implementation. This step allows researchers to gather feedback from experts and children for further improvements [20,59]. A pilot test, followed by a clinical trial, is recommended, which quantitatively analyzes how the prototype achieves the intended outcomes and qualitatively explores users’ experiences. An RCT study design is preferred due to its high quality. The ability to perform a task at an expected level and with minimal adverse events may be set as the intended outcome. It should be carefully determined how long participants will be engaged in the game, how many sessions a child needs to reach the goals, and how long it takes to complete a session. A short duration is associated with unfamiliarity with the tasks, whereas a long duration leads to boredom [60].

Moreover, health outcomes, including clinical, humanistic, and economic outcomes, should be periodically evaluated [61]. Clinical outcomes may include symptom improvement and reduction of morbidity, whereas humanistic outcomes may include knowledge and attitudes, behavior changes, and an improved mindset. As no economic outcomes were available in the studies in this review, researchers are encouraged to evaluate economic outcomes when using the prototype. Possible unexpected impacts of SG interventions on aggressive behaviors should also be evaluated, especially for SG interventions that encompass violent elements [62,63]. Continued discussions with clinicians are still relevant to ensure that the game world setting can be applied to the real world.

##### Marketing and Monitoring Use of the Prototype (Step 6)

Disseminating and promoting a well-evaluated SG educational tool can enhance access to a broader population that may benefit the most and promptly inform the game industry to invest in such interventions. Commercialization of an SG educational tool for children remains a challenge due to the need for high-end technologies, animated multimedia design, artists, illustrators, and other consoles. Gameplay is rapidly changing due to advances in technology, and it should be developed in line with current modalities to minimize the obsolescence of software and hardware [39]. To ensure market readiness, business experts should be consulted and involved throughout the process. It is recommended for researchers to accelerate partnerships with the gaming industry for sustainable SG maintenance.

Specific practice skills can be designed in a modest simulation. For example, children with type 1 diabetes should be able to use insulin regularly, exercise, maintain a healthy diet, and be aware of the signs of hypoglycemia. Modest instruction will help clinicians in applying SG education for children with chronic diseases in the real world. It is important to underline that an excellent performance in the game world is not directly associated with mastery in the real world. Practicing self-management skills, such as physical activities and medication use, should regularly be guided by health care professionals. It may be relevant to consult with policy makers and health care associations regarding the establishment of policies and recommendations for appropriate uses of SG educational tools in clinical practice. Postmarketing feedback should continually be collected to improve the SG’s quality.

## Discussion

### Principal Findings

This scoping review proposed a digital framework to design SG educational tools for children with chronic diseases. The SG framework consists of 3 main strategies to guide the planning, design and development, and implementation of SG educational tools to allow children to practice self-management skills for their chronic condition. Major considerations of how each step is conceptualized, including a theory-driven foundation, contents of health education, joyful reinforcement, and use of technology, were discussed. The game elements and game structure should engage children’s attention and support them in performing gamified self-management tasks, changing their mindset, and increasing their self-care abilities.

### Comparison With Prior Work and Considerations for Using the Proposed Framework

Implementation of SG educational tools for children with chronic diseases has been demonstrated in several previous works [8,9,58], specifically concerning health education [55,64], physical activities [65,66], and self-management [9,67,68]; however, none of them offer a theory-driven framework for behavior change. It has been suggested that researchers articulate a scientific framework for the design of SG educational tools [65]. Although behavioral and self-management interventions can be delivered to children from 5 to 18 years of age [67,68], the health educational content is not applicable to the entire age range. Educational materials for children should be supplemented with communication skills, whereas activities for adolescents should focus on self-monitoring and problem-solving [69]. Multidisciplinary teamwork from conception to marketing is strongly emphasized [64], which was accommodated in this framework throughout the proposed phases.

As game-based interventions are continually growing, researchers are considering developing SG educational tools for children, but questions about how to get started have been raised. Developing an SG educational tool is expensive; therefore, several aspects should be carefully considered before initiating development of SG educational tools, including securing funding and building a collaborative team [69]. Developing SG educational tools for children with chronic diseases differs from entertainment-only video games due to their unique components of behavioral theories and learning materials to boost self-management practices and promote positive behavior changes. For example, children with asthma may need knowledge about preventing asthma triggers and adhering to medication, whereas children with cystic fibrosis may need more physical rehabilitation activities than children with other chronic respiratory diseases. Some of them may need specific, scheduled physical activities, whereas others may need the efforts of encouragement or psychological support and companionship. That is why the game design should be able to address those needs.

Establishing a solid team, which involves experienced game developers or game companies, should be noted. Once members are chosen, clear ground responsibilities and expectations regarding the prototype design should be established. The health care professional team can develop appropriate health learning contents and discuss those materials with the game developer team to analyze and resolve potential problems before programming and prototyping. As there is no reimbursement for SG use as a medical treatment [69], acquiring available funding and resources should be prioritized.

### Challenges and Pitfalls of SG Design and Development

Developing appropriate SG educational tools for the specific needs of children with chronic diseases remains a challenge due to the huge investment from ideas to implementation. As the market for SG-based interventions expands across health conditions, there is a trend for SG education to be included as a supportive intervention rather than merely as pure entertainment [23].

On the basis of our heterogeneous results, the procedure through which SG educational tools deliver content might not be the only key contributor to achieve the targeted goals because the intervention should be focused not only on the learning materials but also on the intertwined mechanism of game elements and the elements of behavioral theory. In the context of game-based learning, self-management practices will be correctly performed if users are enjoying themselves, which means having the propensity to engage, blend, and learn. From this perspective, we raised several considerations on the potential of game elements to enhance intrinsic motivation, including how much autonomy (videos, animations or cartoons, choices, and difficulty adjustments of the challenges) must be given to children during play, how can relatedness (narrative or storyline, avatars, characters, and tailored messages) between children and the game be built into SG educational tools, and how can a child’s level of competence be defined to challenge them.

Several critical points in each step were pointed out for game designers to avoid failure. First, there can be failure to define suitable educative materials and targeted behaviors for children with specific difficulties. Second, one can fail to generate a dynamic between players and the game while, at the same time, players have to obtain new learning from the SG educational tools. Game levels were revealed to engage players with a positive learning effect; however, this should be in line with the player’s skills and cognitive development. A high-challenge game with low-skill, fixed-mindset users may induce frustration; meanwhile, a low-challenge game for users with high skills and a growth mindset may generate feelings of triviality [6]. Given rapid trends in digital technology, SG prototypes should be continually adjusted to prevent them from becoming hackneyed by the time the evaluation trial is finished.

### Safety Concerns for Children in the Post–COVID-19 Era

Safety aspects of SG educational tools should be of general concern because these tools are considered a persuasive technology for changing human behaviors. Game-based interventions appear to be most effective in users aged <18 years [23]; nevertheless, children and adolescents vary in their ability to master a mission. Children may feel engaged with customizable avatars, but some of these may contain violent characters [19]. Game designers should ensure that the SG intervention is not dangerous or does not increase risks to children, such as by promoting sedentary or aggressive behaviors [47] or increasing the risk of physical injuries due to practicing skills. Several harmful risks are associated with sleep disorders and internet gaming disorders, such as anxiety, unsuccessful attempts at control, and jeopardizing environments [19,70].

The American Academy of Pediatrics has stated concerns about the influence of digital media on the health and cognitive development of children at the ages of 0 to 5 years, and it has proposed limiting screen use to 1 hour per day for children aged 2 to 5 years [71]. It is also recommended to avoid screen time 1 to 2 hours before bedtime for children and adolescents. In 2020, the American Academy of Ophthalmology recommended the 20-20-20 rule, described as a 20-second break every 20 minutes by looking 20 feet away to prevent and relieve digital eyestrain [72].

The COVID-19 pandemic intensified gaming behaviors among children, especially during school closures, and this garnered the concern of policy makers and health care professionals [15]. Sedentary time in children with chronic diseases might have increased [73] as parents were not well prepared for it due to their attention being focused on social and economic burdens caused by the pandemic. Several SG educational tools were developed during the pandemic to stimulate in-home physical rehabilitation [74,75] and improve anxiety and mood disorders in adolescents [76], and those positive behavioral outcomes should be maintained. Even though the pandemic situation has improved, some parents are continuing to work remotely while simultaneously caring for children, leading to obstacles to maintaining children’s learning, especially in households of a low socioeconomic status [77].

Educational, game-based interventions for the post–COVID-19 era should be integrated with appropriate recommendations for their use. Individualized family media use plans are strongly recommended; hence, parental control is central when exposing children to digital media [70]. It is considered important for parents to accompany their children during screen use to foster an effective learning process by understanding the game structure, supporting children in controlling playing times, and monitoring their activities. Instead of giving punishment as a disciplinary matter, by playing together, parents can understand more about SG educational tools and how they can facilitate parent-child interactions. As parents become familiar with their children’s games, they will be able to encourage their children to achieve the intended outcomes and avoid addictive behaviors [78].

### Limitations

This scoping review has a few limitations. This framework was developed based on a review of the most relevant SG educational tools in several RCTs and SG framework studies instead of a direct participatory approach involving health care professionals and children. When comparing the effects of SG educational tools, most RCTs (9/16, 56%) only captured improvements in humanistic outcomes, such as knowledge [57] and enjoyment. Studies on improving clinical outcomes were limited, and none provided economic outcome evaluations. This is in line with the findings of a previous review that presented a lack of clinical evidence of the implementation of SG educational tools in children with neurodevelopmental disorders [79]. Several studies (5/16, 31%) evaluated changes in knowledge over a relatively short duration on beneficial effects on behaviors. Exploration is still needed as to which game elements can have higher effects on self-management and behavior changes. Moreover, issues of maintenance of intended behaviors after exposure to SG interventions should be carefully addressed. With the limitations of the available literature, this framework should be tested in further studies.

### Implications of the Study and Further Research

This framework provides a theory-driven step-by-step approach to help health educators, clinicians, game developers, and policy makers more efficiently develop SG educational tools for children with chronic diseases. Understanding how to integrate the power of SG educational tools offers significant promise for promoting health behavior changes. Only 4% of the top-rated health apps apply the concepts of gamification [80], indicating that the opportunity to develop high-quality SG educational tools for children with chronic diseases is still wide open. Further research should explore the needs for culture-specific SG educational tools and investigate the mediators of behavior change.

### Conclusions

A framework of SG-based educational tools promoting self-management activities and behavior changes in children with chronic diseases was developed by incorporating behavioral principles and mechanisms of SGs. It expedites the translation of fundamental behavioral theories and game elements into a scaled-up industrial level in which digital-based game interventions are being created to enhance children’s participation and motivation. The effectiveness of SG educational tools in achieving targeted behaviors depends on key designs and elements of how they address problems and mindsets of children with difficulties. Underpinning appropriate behavioral theories, learning materials, game elements, esthetics, and technology should be considered in all phases of research. The design, development, and evaluation of SG educational tools for children with chronic diseases need to be broadly explored with the support of a well-validated game-based framework and the deployment of advanced technologies.

## Appendix

Multimedia Appendix 1 Supplementary tables and figures.

Multimedia Appendix 2 Preferred Reporting Items for Systematic reviews and Meta-Analyses extension for Scoping Reviews (PRISMA-ScR) Checklist.

## Abbreviations

CMO: context-mechanism-output
PRISMA-ScR: Preferred Reporting Items for Systematic Reviews and Meta-Analyses extension for Scoping Reviews
RCT: randomized controlled trial
SDT: self-determination theory
SG: serious game
