# Supplementary material for: Digital Serious Games to Promote Behavior Change in Children With Chronic Diseases: Scoping Review and Development of a Self-Management Learning Framework
Source: J Med Internet Res. 2024 Aug 19;26:e49692. doi: 10.2196/49692 (PMC11369548; doi:10.2196/49692)
Supplement: Multimedia Appendix 1 [file jmir_v26i1e49692_app1.docx]

**Multimedia Appendix 1.**

**Title:** Digital Serious Games to Promote Behavioral Change in Children with Chronic Diseases: Review and Development of a Self-Management Learning Framework

**Table S1.** The query of the search strategy in the PubMed database

|  | **Query** |
| --- | --- |
| 1 | serious game[Title/Abstract],,,"""serious game""[Title/Abstract]" |
| 2 | computer game[Title/Abstract],,,"""computer game""[Title/Abstract]" |
| 3 | web-based game[Title/Abstract],,,"""web based game""[Title/Abstract]" |
| 4 | internet game[Title/Abstract],,,"""internet game""[Title/Abstract]" |
| 5 | mobile game[Title/Abstract],,,"""mobile game""[Title/Abstract]" |
| 6 | video game[Title/Abstract],,,"""video game""[Title/Abstract]" |
| 7 | gamification[Title/Abstract],,,"""gamification""[Title/Abstract]" |
| 8 | ((((((serious game[Title/Abstract]) OR (computer game[Title/Abstract])) OR (web-based game[Title/Abstract])) OR (internet game[Title/Abstract])) OR (mobile game[Title/Abstract])) OR (video game[Title/Abstract])) OR (gamification[Title/Abstract]),,,"""serious game""[Title/Abstract] OR ""computer game""[Title/Abstract] OR ""web based game""[Title/Abstract] OR ""internet game""[Title/Abstract] OR ""mobile game""[Title/Abstract] OR ""video game""[Title/Abstract] OR ""gamification""[Title/Abstract]" |
| 9 | self-management[Title/Abstract],,,"""self-management""[Title/Abstract]" |
| 10 | behaviour change[Title/Abstract],,,"""behaviour change""[Title/Abstract]" |
| 11 | chronic disease[Title/Abstract],,,"""chronic disease""[Title/Abstract]" |
| 12 | chronic illness[Title/Abstract],,,"""chronic illness""[Title/Abstract]" |
| 13 | (((self-management[Title/Abstract]) OR (behaviour change[Title/Abstract])) OR (chronic disease[Title/Abstract])) OR (chronic illness[Title/Abstract]),,,"""self-management""[Title/Abstract] OR ""behaviour change""[Title/Abstract] OR ""chronic disease""[Title/Abstract] OR ""chronic illness""[Title/Abstract]" |
| 14 | child[Title/Abstract]" OR "children[Title/Abstract]" |
| 15 | ((((((((serious game[Title/Abstract]) OR (computer game[Title/Abstract])) OR (web-based game[Title/Abstract])) OR (internet game[Title/Abstract])) OR (mobile game[Title/Abstract])) OR (video game[Title/Abstract])) OR (gamification[Title/Abstract])) AND ((((self-management[Title/Abstract]) OR (behaviour change[Title/Abstract])) OR (chronic disease[Title/Abstract])) OR (chronic illness[Title/Abstract]))) AND (children[Title/Abstract]),,,"(""serious game""[Title/Abstract] OR ""computer game""[Title/Abstract] OR ""web based game""[Title/Abstract] OR ""internet game""[Title/Abstract] OR ""mobile game""[Title/Abstract] OR ""video game""[Title/Abstract] OR ""gamification""[Title/Abstract]) AND (""self-management""[Title/Abstract] OR ""behaviour change""[Title/Abstract] OR ""chronic disease""[Title/Abstract] OR ""chronic illness""[Title/Abstract]) AND ""children""[Title/Abstract]" |
| 16 | (((((((serious game[Title/Abstract]) OR (computer game[Title/Abstract])) OR (web-based game[Title/Abstract])) OR (internet game[Title/Abstract])) OR (mobile game[Title/Abstract])) OR (video game[Title/Abstract])) OR (gamification[Title/Abstract])) AND (children[Title/Abstract]),,,"(""serious game""[Title/Abstract] OR ""computer game""[Title/Abstract] OR ""web based game""[Title/Abstract] OR ""internet game""[Title/Abstract] OR ""mobile game""[Title/Abstract] OR ""video game""[Title/Abstract] OR ""gamification""[Title/Abstract]) AND ""children""[Title/Abstract]" |
| 17 | (((((((serious game[Title/Abstract]) OR (computer game[Title/Abstract])) OR (web-based game[Title/Abstract])) OR (internet game[Title/Abstract])) OR (mobile game[Title/Abstract])) OR (video game[Title/Abstract])) OR (gamification[Title/Abstract])) AND (children[Title/Abstract]),,,"(""serious game""[Title/Abstract] OR ""computer game""[Title/Abstract] OR ""web based game""[Title/Abstract] OR ""internet game""[Title/Abstract] OR ""mobile game""[Title/Abstract] OR ""video game""[Title/Abstract] OR ""gamification""[Title/Abstract]) AND ""children""[Title/Abstract]" |
| 18 | (children[Title/Abstract]) OR ((((self-management[Title/Abstract]) OR (behaviour change[Title/Abstract])) OR (chronic disease[Title/Abstract])) OR (chronic illness[Title/Abstract])),,,"""children""[Title/Abstract] OR ""self-management""[Title/Abstract] OR ""behaviour change""[Title/Abstract] OR ""chronic disease""[Title/Abstract] OR ""chronic illness""[Title/Abstract]" |
| 19 | (((((((serious game[Title/Abstract]) OR (computer game[Title/Abstract])) OR (web-based game[Title/Abstract])) OR (internet game[Title/Abstract])) OR (mobile game[Title/Abstract])) OR (video game[Title/Abstract])) OR (gamification[Title/Abstract])) AND ((children[Title/Abstract]) OR ((((self-management[Title/Abstract]) OR (behaviour change[Title/Abstract])) OR (chronic disease[Title/Abstract])) OR (chronic illness[Title/Abstract]))),,,"(""serious game""[Title/Abstract] OR ""computer game""[Title/Abstract] OR ""web based game""[Title/Abstract] OR ""internet game""[Title/Abstract] OR ""mobile game""[Title/Abstract] OR ""video game""[Title/Abstract] OR ""gamification""[Title/Abstract]) AND (""children""[Title/Abstract] OR (""self-management""[Title/Abstract] OR ""behaviour change""[Title/Abstract] OR ""chronic disease""[Title/Abstract] OR ""chronic illness""[Title/Abstract]))" |

**Table S2.** Identifying SG components for the proposed SG framework (*N*=30)

|  | **Included SG framework studies (*n*=14)** | **Included randomized clinical studies (*n*=16)** |
| --- | --- | --- |
| **Context** | |  |
| Behavioral theories | [1-13] | [2, 14-23] |
| Game theories | [1-8, 10-13, 24] | [2, 14-19, 23] |
| Educational materials | [1-13, 24] | [2, 14-23, 25] |
| **Mechanism** | |  |
| Game goals | [1-13, 24] | [2, 14-23, 25-29] |
| Game genre, rules | [2-5, 8, 11-13, 24, 30] | [2, 14-21, 23, 25, 26, 28] |
| Aesthetics (images, sound, graphics) | [1-13, 24] | [2, 14-17, 21-23, 25-28] |
| Personalization (characters, avatars) | [1-6, 8-13] | [2, 14-23] |
| Narrative (storyline) | [1-5, 7-13, 24] | [2, 14-23] |
| Challenges | [1-6, 8-13] | [2, 14-23, 26-29] |
| Feedback (tailored messages) | [1-13] | [2, 14-23, 25-29] |
| Rewards (points, scores) | [1-13] | [2, 14-23, 27, 28] |
| **Outcomes** | |  |
| Health outcomes  Behavioral outcomes  Learning outcomes  Clinical outcomes |  | [2, 14-21, 23, 25-28]  [2, 14-23, 25]  [2, 14-16, 18, 19, 21, 26, 28, 29] |
| Dissemination of the SG framework  A specific framework for children  General framework | [1-6, 8, 10]  [7, 9, 11-13, 24] |  |

**Table S3.** Game elements on behaviour change within in current available game frameworks.

| **Authors, year** | **Game Elements** |
| --- | --- |
| AlMarshedi et al, 2016 ^[1]^ | Visualisation, Motivation, Socializing, Self-representation, Fun, Esteem, Growth, Sustainability. |
| Baranowski et al, 2011 ^[2]^ | Exposure, Simulation, Feedback, Knowledge, Story, Self-control, Tailored motivational messages, Behavioural inoculation. |
| Dorrenbacher et al, 2014 ^[5]^ | Story, Layout, Rewards, Feedback, Motivation, Choice, Presence, Challenge, Autonomy, Training. |
| Hansen et al, 2017 ^[6]^ | Avatars, Badges, Challenges, Leader boards, Levels, Progress bars, Rewards, Social interaction. |
| Starks et al, 2018 ^[13]^ | Social cognitive, Intelligence, Enjoyment. |
| Thompson et al, 2011 ^[30]^ | Intervention, Mediators, Autonomy, Mastery, Self-efficacy, Competence, Motivation, Relatedness. |

**Table S4.** Three Domains of the Self-Determination Theory

| **Domain** | **Components** |
| --- | --- |
| Competence | Challenges, learning materials, tasks, instructions, tutorials, demonstrations, competitions, levels, progress bars |
| Autonomy | Choices, control, visual presentations, audio and sounds, stories, avatars, characters, difficulty adjustments |
| Relatedness | Feedback, messages, help-button, motivation, emotions, social support |

**Table S5.** Game taxonomy

| **Category** | **Components** |
| --- | --- |
| Social | In-game voice, text chat, internet forum, social media forum/group, strategy guide, tailored messages, feedback, motivational call-out, help button |
| Presentation | Realistic graphics, dimension graphics, music, sounds, videos, images, logos, cartoons, characters speaking, interface/layout presentation |
| Narrative and identity | Choice of sex, choice of attributes/clothes/avatar/role model, choice of colors, choice of stories, cut-scenes, mission briefing, role-playing, movements |
| Rewards and punishments | Points, bonuses, stars, badges, incentives, progress bar, trend alerts, losing a life, restarting a level, level of difficulty, and unlimited replayability of the game |
| Manipulation and control | Choice of challenges, checkpoints, competition with others (multiplayer option) |

**Table S6.** Identifying behavioral theories from all included studies (*N*=29)

|  | **Behavioral theories** | | | | | | **Learning theories** | | | **Game theories** | | | **Other theories** |
| --- | --- | --- | --- | --- | --- | --- | --- | --- | --- | --- | --- | --- | --- |
|  | **SDT** | **ELM** | **FT** | **TT** | **UCD** | **HBM** | **SLT** | **SCT** | **PPM** | **MDA** | **LM-GM** | **ToIA** |  |
| AlMarshedi et al. (2016) * | + |  | + |  |  |  |  |  |  |  |  |  | SGI |
| Baranowski et al. (2011) * | + | + |  | + |  |  |  | + |  |  |  | + |  |
| Carvalho et al. (2015) |  |  |  |  | + |  |  |  |  | + | + |  | HABS, GOP, FDF, FTCG |
| Colorado et al. (2021) | + |  | + |  | + |  |  |  |  |  | + |  | PROGAME, HUFIT, UML, GDFEBCPL |
| Dorrenbacher et al. (2014) * | + |  |  |  |  |  |  |  |  | + |  |  |  |
| Epstein et al. (2021) |  |  |  |  |  |  |  |  |  | + |  |  |  |
| Hansen (2017) * | + |  |  |  | + |  |  |  |  |  |  |  | LISA |
| Jaccard et al. (2021) |  |  |  |  |  |  |  |  |  |  | + |  | SERES, SFSGD, DISC, SGAF, TFSG, CFSG |
| Khaleghi et al. (2021) * | + |  | + |  | + |  |  |  |  |  |  |  | DSR methodology, ASMEM, OGF |
| Mummah et al. (2016) |  |  |  |  | + | + |  | + |  |  |  |  | HBM, BCT, HITUEM, |
| Stark (2014) |  |  |  |  |  |  |  | + |  |  |  |  | MI, Enjoyment |
| Thompson et al. (2011) * | + | + |  | + |  |  |  | + |  |  |  | + |  |
| Verschueren et al. (2019) |  |  |  |  |  |  |  |  |  |  | + |  | Agile scrum, TGL, CBGD, TSSGH |
| Wattanasoontorn et al. (2013) |  |  |  |  |  |  |  |  |  |  |  |  | ToHL |
| Baranowski et al. (2011) * | + | + |  | + |  |  |  | + |  |  |  | + | MMVM |
| Baranowski et al. (2019) * | + | + |  | + |  |  |  | + |  |  |  | + | MMVM |
| Brown et al. (1997) |  |  |  |  |  |  |  |  |  |  |  |  | HCI |
| Hamari et al. (2019) |  |  |  |  |  |  |  |  |  |  | + |  |  |
| Kato et al. (2008) |  |  |  |  |  |  | + | + |  |  |  |  |  |
| Pin et al. (2019) |  |  |  |  |  |  |  |  |  |  | + |  |  |
| Salonini et al. (2015) |  |  |  |  |  |  |  |  |  |  |  |  |  |
| Bartholomew et al. (2000) |  |  |  |  |  |  |  | + |  |  |  |  |  |
| Gomes et al. (2015) |  |  |  |  |  |  |  |  |  |  | + |  |  |
| Huss et al. (2003) |  |  |  |  |  |  | + |  | + |  |  |  |  |
| McPherson et al. (2005) |  |  |  |  |  |  |  |  |  |  | + |  |  |
| Rubin et al. (1986) |  |  |  |  |  |  | + |  |  |  |  |  |  |
| Shames et al. (2004) |  |  |  |  |  |  |  |  |  |  | + |  |  |
| Yawn et al. (2000) |  |  |  |  |  |  |  |  |  |  | + |  |  |
| Winskell et al. (2018) |  |  |  |  |  |  |  | + |  |  |  |  | The theory of possible selves |
| **TOTAL** | **9*** | **4** | **3** | **4** | **5** | **1** | **3** | **9** | **1** | **3** | **10** | **4** |  |

*Included in the analysis (*n*=9).

ASMEM: Assembly-based Situational Method Engineering Methodology; BCT: Behaviour Change Technique; CFSG: Conceptual Framework for Serious Game; DISC: The DISC Model; DSR: Design Science Research; ELM: Elaboration Likelihood Model; FDF: Four Dimensional Framework; FT: Flow Theory; FTCG: Flow Theory of Computer Game; GOP: Game Ontology Project; GDFEBCPL: Game Design Framework for Evidence-Based Clinical Procedure Libraries; HABS: Hierarchical Activity-Based Scenario; HBM: Health Belief Model; HCI: Human-Computer Interaction; HITUEM: Health Information Technology Usability Evaluation Model; HUFIT: the HUFIT planning, analysis and specification toolset; IDEA: Integrate, Design, Assess, and Share; LISA: Lens of Intrinsic Skill Atoms; LM-GM: Learning Mechanics-Game Mechanics; MDA: Mechanics, Dynamics, Aesthetics; MI: Multiple Intelligences; MMVM: Mediating Moderating Variable Model ; OGF: Octalysis Gamification Framework; PPM: the Precede-Proceed Models; PROGAME: the PROGAME Framework for Motor Rehabilitation Therapy; SDT: Self-Determination Theory; SCT: Social-Cognitive Theory; SLT: Social-Learning Theory; SERES: The SERES Framework; SFSGD: Six Facets of Serious Game Design; SGAF: Serious Game Assessment Framework; SGI: Sustainable Gamification Impact; TGL: Theory of Gamified Learning; TFSG: Theoretical Framework for Serious Game; ToIA: Theory of Immersion and Attention; ToHL: Theory of Homo-Ludens; TSSGH: Three Steps of Serious Game for Health; TT: Transportation Theory; UCD: User Centered Design; UML: The Unified Modeling Language Reference.

**Table S7.** Identifying game elements from all included studies (*N*=30)

|  |  | **Social** | **Presentation** | **Narrative and identity** | **Rewards and punishments** | **Manipulation and control** |
| --- | --- | --- | --- | --- | --- | --- |
| 1 | AlMarshedi et al. (2016) [1]* | Feedback, community, and social media support | Visualization, trend alerts | Avatars, story, theme | Challenges, rewards (badges and points) | Self-management tasks, log-book, goals, tiny habits, levels, competition, progress bar, leader boards |
| 2 | Baranowski et al. (2011) [2]* | Feedback, tailored messages, social support | Video images, bars, sounds, specific devices, fun | Story, plot, character, emotion | Challenges, rewards (incentives and points) | Levels, flow, competition |
| 3 | Carvalho et al. (2015) [3] | Feedback, messages, reports | Information, tutorial, tiles, user interface | Avatars | Challenges, performance meter, rewards | Customization, review repetition, imitating, multiple chances, choices, levels |
| 4 | Colorado et al. (2021) [4] | Feedback | Accessibility (user interface) | Avatars | Challenges, rewards | Mastery, adaptability, real-life simulation, concentration, difficulty adjustment |
| 5 | Dorrenbacher et al. (2014) [5]* | Feedback | Stimulus material, instructions, layout | Story, presence | Challenges, rewards | Categorization of tasks, training, goals, motivation, choices, autonomy |
| 6 | Epstein et al. (2021) [24] | Feedback, social support | Aesthetic (user interface), artwork | Storyline, emotions | Challenges, rewards, punishments | Competition, roles |
| 7 | Hansen (2017) [6]* | Feedback, social support | Application, user interface | Avatars | Challenges, rewards (achievements, badges, points) | Progression, competition, leader boards, levels |
| 8 | Jaccard et al. (2021) [7] | Feedback | Profile, functions, objectives, rules, structure, interface, context, game outline | Pedagogical scenario, narrative, user experience | Rewards (incentives) | Goals, fidelity, simulation, flow |
| 9 | Khaleghi et al. (2021) [8]* | Feedback | Images, audio, videos, animations | Avatars, emotions | Challenges, rewards | Leader boards, tasks, levels, rules |
| 10 | Mummah et al. (2016) [9] | Feedback | Context, interface, sounds, colors | Identity, personalization, avatars, tastes, story, characters | Challenges, rewards | Choice/control, competition, growth mindset, activity levels |
| 11 | Stark (2014) [13] | Feedback, role model | Graphics, music/sounds, humor, math/numbers, words/language | Narrative, story, logic/patterns, personal reflection, role models, cartoons | Challenges, rewards (incentives) | Physical movements, levels, objectives, flow, choices, engagement (immersion) |
| 12 | Thompson et al. (2011) [10]* | Feedback | Music, sounds, cartoons, user interface | Story, characters | Challenges, rewards | Goals, problem-solving, review, choices, mastery |
| 13 | Verschueren et al. (2019) [11] | Feedback | Game platform, game authoring tool, engine, database, data protection, objective, content, visual interface, aesthetic, graphics | Storyline, genre, narrative | Challenges, rewards (badges, points) | Self-management tasks, difficulty adjustment, problem-solving, rules |
| 14 | Wattanasoontorn et al. (2013) [12] | Feedback | Interface, engine, platform, tools/special equipment, objective | Avatars/ model | Challenges, rewards | Progress bar, levels |
| 15 | Baranowski et al. (2011) [2]* | Feedback, supportive messages | Cartoons, sounds | Genre, story, characters | Challenges, rewards | Levels with difficulties |
| 16 | Baranowski et al. (2019) [14]* | Feedback | Cartoons, sounds | Genre, story, characters | Challenges, rewards | Levels with difficulties |
| 17 | Bartholomew et al. (2000) [15] | Feedback, records | Cartoons, sounds | Genre, story, characters | Challenges | Self-management tasks, options, problem-solving |
| 18 | Brown et al. (1997) [16] | Feedback, logbook | Cartoons, sounds | Genre, story, characters | Challenges, rewards | Self-management tasks, levels, options/choices |
| 19 | Gomes et al. (2015) [26] | Feedback | Adventure interface, sounds | Genre, instructions | Challenges, rewards | Physical tasks, levels |
| 20 | Hamari et al. (2019) [27] | Feedback, phone call | Cartoons, sounds, specific tools | Genre, instructions | Challenges | Physical tasks |
| 21 | Huss et al. (2003) [17] | Feedback, on-screen providers | Cartoons, sounds | Genre, story, characters | Challenges | Self-management tasks, levels with difficulties |
| 22 | Kato et al. (2008) [18] | Feedback | Cartoons, sounds | Genre, story, characters | Challenges, rewards | Options, players control a 3-dimensional environment, chances, levels |
| 23 | McPherson et al. (2005) [19] | Feedback, records | Cartoons, sounds | Genre, story, characters | Challenges | Self-management tasks |
| 24 | Pin et al. (2019) [29] | Feedback | Adventure interface, sounds, equipment | Genre, instructions | Challenges | Physical tasks, levels |
| 25 | Rubin et al. (1986) [20] | Feedback, records | Cartoons, sounds | Genre, story, simulations | Challenges, rewards (incentives) | Self-management tasks, options/choices |
| 26 | Salonini et al. (2015) [28] | Feedback | Adventure interface, sounds | Genre, instructions | Challenges, rewards (points) | Physical tasks, levels |
| 27 | Shames et al. (2004) [21] | Feedback, case manager, hotline access | Cartoons, sounds | Genre, story, characters, models | Challenges, rewards (incentives) | Self-management tasks, levels |
| 28 | Yawn et al. (2000) [22] | Feedback, symptoms log book, dictionary | Cartoons, sounds | Genre, story, characters, scenario, model | Challenges | Self-management tasks, levels |
| 29 | Winskell et al. (2019) [23] | Feedback, supportive messages | Cartoons, sounds | Genre, story | Challenges, rewards | Options/choices |
| 30 | Weiland et al. (2022) [25] | Feedback | Cartoons, sounds | Genre, instruction | Challenges | Self-management tasks |

* Included in the analysis (*n*=9).

**Table S8.** Intersecting the Self-Determination Theory domains and game elements (*n*=9)

| **Self-Determination Theory** | **Game taxonomy** | | | | |
| --- | --- | --- | --- | --- | --- |
|  | **Social** | **Presentation** | **Narrative and identity** | **Rewards and punishments** | **Manipulation and control** |
| *AlMarshedi, 2016 [1]* | | | | | |
| Competence |  |  |  | Progress bar, points | Challenges, competition, levels, self-management tasks |
| Autonomy |  | Images, video |  |  |  |
| Relatedness | Feedback,  social media |  | Avatars, story, theme |  |  |
| *Baranowski, 2011 [2];*  *Baranowski, 2019 [14]* | | | | | |
| Competence |  |  |  | Points, stars | Challenges, competition, game rules, goals, levels |
| Autonomy |  | Images, videos |  |  | Difficulty adjustments |
| Relatedness | Feedback,  tailored messages |  | Characters, emotions, story |  |  |
| *Colorado, 2021 [4]* | | | | | |
| Competence |  | Materials, tutorials |  | Badges | Challenges |
| Autonomy |  | Attractive layout |  |  | Difficulty adjustments |
| Relatedness | Feedback |  | Avatars |  |  |
| *Dorrenbacher, 2014 [5]* | | | | | |
| Competence |  | Instructions |  | Badges, stars | Challenges, goals, tasks |
| Autonomy |  | Attractive layout |  |  | Choices |
| Relatedness | Feedback |  | Story, simulations |  | Motivation |
| *Hansen, 2017 [6]* | | | | | |
| Competence |  |  |  | Badges, stars, progress bar | Challenges, competition, levels |
| Autonomy |  | Attractive layout |  |  |  |
| Relatedness | Feedback,  social media |  | Avatars |  |  |
| *Khaleghi, 2021 [8]* | | | | | |
| Competence |  |  |  | Points, badges | Challenges, game rules, levels, tasks |
| Autonomy |  | Animation, images, videos |  |  |  |
| Relatedness | Feedback |  | Avatars, emotions |  |  |
| *Thompson, 2011 [10]* | | | | | |
| Competence |  |  |  | Points, stars | Challenges, goals, tasks |
| Autonomy |  |  |  |  | Choices |
| Relatedness | Feedback | Cartoons, music, sounds | Story, characters |  |  |

**Figure S1.** Game structure of the serious game (SG) prototype.

**Autonomy**

**Self-Determination Theory**

**Competence**

No skill achieved

**Achieve the rewards, evaluate the performance:**

- Skill performance
- Feedback
- Reflection

**Choose the preferences:**

- Learning: Narrative
- Gaming: Avatars, characters, emotions

**Observe the introduction:**

- Learning:

Learning materials

- Gaming: Game rules, instructions

**Play:**

Game sessions

**Start the prototype**

**Relatedness**

Achieve new skill

Goals

Learn the game instruction

Understand how to complete the tasks

Complete the tasks

Mastery, behavior change

**Learning**

Action

Choose the narrative

Learn the educative materials

Demonstrate the abilities

Evaluate the performance

Elements

Narrative plots

Narrative/ story

Questions, reminders

Performance meter

Goals

Configure the game

Understand how to play

Engage with the game

Motivation, engagement

**Gaming**

Action

Choose the preferences

Learn game instructions

Take the challenges

Evaluate the performance

Game elements

Elements

Avatars or characters

Game instruction

Rewards, score

**References**

1. AlMarshedi, A., G. Wills, and A. Ranchhod, *Guidelines for the Gamification of Self-Management of Chronic Illnesses: Multimethod Study.* JMIR Serious Games, 2017. **5**(2): p. e12.

2. Baranowski, T., et al., *Video game play, child diet, and physical activity behavior change a randomized clinical trial.* Am J Prev Med, 2011. **40**(1): p. 33-8.

3. Carvalho, M.B., et al., *An activity theory-based model for serious games analysis and conceptual design.* Computers & Education, 2015. **87**: p. 166-181.

4. Beristain-Colorado, M.D.P., et al., *Standardizing the Development of Serious Games for Physical Rehabilitation: Conceptual Framework Proposal.* JMIR Serious Games, 2021. **9**(2): p. e25854.

5. Dörrenbächer, S., et al., *Dissociable effects of game elements on motivation and cognition in a task-switching training in middle childhood.* Front Psychol, 2014. **5**: p. 1275.

6. Hansen, O.G. *What gamification design do users want in a self-management application for chronic diseases? - The case of Cystic Fibrosis*. 2017.

7. Jaccard, D., et al., *The co.LAB Generic Framework for Collaborative Design of Serious Games: Development Study.* JMIR Serious Games, 2021. **9**(3): p. e28674.

8. Khaleghi, A., Z. Aghaei, and M.A. Mahdavi, *A Gamification Framework for Cognitive Assessment and Cognitive Training: Qualitative Study.* JMIR Serious Games, 2021. **9**(2): p. e21900.

9. Mummah, S.A., et al., *IDEAS (Integrate, Design, Assess, and Share): A Framework and Toolkit of Strategies for the Development of More Effective Digital Interventions to Change Health Behavior.* J Med Internet Res, 2016. **18**(12): p. e317.

10. Thompson, D., *Designing serious video games for health behavior change: current status and future directions.* J Diabetes Sci Technol, 2012. **6**(4): p. 807-11.

11. Verschueren, S., C. Buffel, and G. Vander Stichele, *Developing Theory-Driven, Evidence-Based Serious Games for Health: Framework Based on Research Community Insights.* JMIR Serious Games, 2019. **7**(2): p. e11565.

12. Wattanasoontorn, V., et al., *Serious games for health.* Entertainment Computing, 2013. **4**(4): p. 231-247.

13. Starks, K., *Cognitive behavioral game design: a unified model for designing serious games.* Front Psychol, 2014. **5**: p. 28.

14. Baranowski, T., et al., *Videogames That Encourage Healthy Behavior Did Not Alter Fasting Insulin or Other Diabetes Risks in Children: Randomized Clinical Trial.* Games Health J, 2019. **8**(4): p. 257-264.

15. Bartholomew, L.K., et al., *Watch, Discover, Think, and Act: evaluation of computer-assisted instruction to improve asthma self-management in inner-city children.* Patient Educ Couns, 2000. **39**(2-3): p. 269-80.

16. Brown, S.J., et al., *Educational video game for juvenile diabetes: results of a controlled trial.* Med Inform (Lond), 1997. **22**(1): p. 77-89.

17. Huss, K., et al., *Computer game for inner-city children does not improve asthma outcomes.* J Pediatr Health Care, 2003. **17**(2): p. 72-8.

18. Kato, P.M., et al., *A video game improves behavioral outcomes in adolescents and young adults with cancer: a randomized trial.* Pediatrics, 2008. **122**(2): p. e305-17.

19. McPherson, A.C., et al., *A randomized, controlled trial of an interactive educational computer package for children with asthma.* Pediatrics, 2006. **117**(4): p. 1046-54.

20. Rubin, D.H., et al., *Educational intervention by computer in childhood asthma: a randomized clinical trial testing the use of a new teaching intervention in childhood asthma.* Pediatrics, 1986. **77**(1): p. 1-10.

21. Shames, R.S., et al., *Effectiveness of a multicomponent self-management program in at-risk, school-aged children with asthma.* Ann Allergy Asthma Immunol, 2004. **92**(6): p. 611-8.

22. Yawn, B.P., et al., *An in-school CD-ROM asthma education program.* J Sch Health, 2000. **70**(4): p. 153-9.

23. Winskell, K., et al., *A Smartphone Game-Based Intervention (Tumaini) to Prevent HIV Among Young Africans: Pilot Randomized Controlled Trial.* JMIR Mhealth Uhealth, 2018. **6**(8): p. e10482.

24. Epstein, D.S., et al., *Tabletop Board Game Elements and Gamification Interventions for Health Behavior Change: Realist Review and Proposal of a Game Design Framework.* JMIR Serious Games, 2021. **9**(1): p. e23302.

25. Weiland, A., et al., *A Serious Game for the Prevention of Obesity in School Children-Impact of Parent's Involvement: A Randomized Controlled Trial.* Life (Basel), 2022. **12**(6).

26. Gomes, E.L., et al., *Active Video Game Exercise Training Improves the Clinical Control of Asthma in Children: Randomized Controlled Trial.* PLoS One, 2015. **10**(8): p. e0135433.

27. Hamari, L., et al., *The effect of an active video game intervention on physical activity, motor performance, and fatigue in children with cancer: a randomized controlled trial.* BMC Res Notes, 2019. **12**(1): p. 784.

28. Salonini, E., et al., *Active Video Game Playing in Children and Adolescents With Cystic Fibrosis: Exercise or Just Fun?* Respir Care, 2015. **60**(8): p. 1172-9.

29. Pin, T.W. and P.B. Butler, *The effect of interactive computer play on balance and functional abilities in children with moderate cerebral palsy: a pilot randomized study.* Clin Rehabil, 2019. **33**(4): p. 704-710.

30. Thompson, D., et al., *Serious Video Games for Health How Behavioral Science Guided the Development of a Serious Video Game.* Simul Gaming, 2010. **41**(4): p. 587-606.
